# Differential effects of selective frankincense (*Ru Xiang*) essential oil versus non-selective sandalwood (*Tan Xiang*) essential oil on cultured bladder cancer cells: a microarray and bioinformatics study

**DOI:** 10.1186/1749-8546-9-18

**Published:** 2014-07-02

**Authors:** Mikhail G Dozmorov, Qing Yang, Weijuan Wu, Jonathan Wren, Mahmoud M Suhail, Cole L Woolley, D Gary Young, Kar-Ming Fung, Hsueh-Kung Lin

**Affiliations:** 1 Arthritis & Clinical Immunology Research Program, Oklahoma Medical Research Foundation, Oklahoma City, OK 73104, USA; 2 Department of Urology, University of Oklahoma Health Sciences Center, Oklahoma City, OK 73104, USA; 3 Department of Physiology, University of Oklahoma Health Sciences Center, Oklahoma City, OK 73104, USA; 4 Dhofar Frankincense Research Plant, Salalah AYUBS42, Sultanate of Oman; 5 Young Living Essential Oils, Lehi, UT 84043, USA; 6 Department of Pathology, University of Oklahoma Health Sciences Center, Oklahoma City, OK 73104, USA; 7 Oklahoma City Veterans Medical Center, Oklahoma City, OK 73104, USA; 8 Department of Urology, University of Oklahoma Medical Center, 920 Stanton L. Young Blvd., WP 3150, Oklahoma City, OK 73034, USA

## Abstract

**Background:**

Frankincense (*Boswellia carterii*, known as *Ru Xiang* in Chinese) and sandalwood (*Santalum album*, known as *Tan Xiang* in Chinese) are cancer preventive and therapeutic agents in Chinese medicine. Their biologically active ingredients are usually extracted from frankincense by hydrodistillation and sandalwood by distillation. This study aims to investigate the anti-proliferative and pro-apoptotic activities of frankincense and sandalwood essential oils in cultured human bladder cancer cells.

**Methods:**

The effects of frankincense (1,400–600 dilutions) (v/v) and sandalwood (16,000–7,000 dilutions) (v/v) essential oils on cell viability were studied in established human bladder cancer J82 cells and immortalized normal human bladder urothelial UROtsa cells using a colorimetric XTT cell viability assay. Genes that responded to essential oil treatments in human bladder cancer J82 cells were identified using the Illumina Expression BeadChip platform and analyzed for enriched functions and pathways. The chemical compositions of the essential oils were determined by gas chromatography–mass spectrometry.

**Results:**

Human bladder cancer J82 cells were more sensitive to the pro-apoptotic effects of frankincense essential oil than the immortalized normal bladder UROtsa cells. In contrast, sandalwood essential oil exhibited a similar potency in suppressing the viability of both J82 and UROtsa cells. Although frankincense and sandalwood essential oils activated common pathways such as inflammatory interleukins (IL-6 signaling), each essential oil had a unique molecular action on the bladder cancer cells. Heat shock proteins and histone core proteins were activated by frankincense essential oil, whereas negative regulation of protein kinase activity and G protein-coupled receptors were activated by sandalwood essential oil treatment.

**Conclusion:**

The effects of frankincense and sandalwood essential oils on J82 cells and UROtsa cells involved different mechanisms leading to cancer cell death. While frankincense essential oil elicited selective cancer cell death *via* NRF-2-mediated oxidative stress, sandalwood essential oil induced non-selective cell death *via* DNA damage and cell cycle arrest.

## Background

Frankincense gum resins (known as *Ru Xiang* in Chinese) are obtained from *Boswellia* trees (family Burseraceae) and have been used for the treatment of rheumatoid arthritis and other inflammatory diseases [1] such as Crohn's disease [2]. Extracts from *Boswellia* species resins exhibit anti-proliferative and pro-apoptotic activities in rat astrocytoma cell lines [3], human leukemia cell lines [4], and chemically induced mouse skin cancer models [5]. The frankincense essential oil possesses anti-proliferative and pro-apoptotic activities against multiple human cancer cell lines *in vitro* and *in vivo*[6-8]. Boswellic acids were found to be the major components in frankincense extracts, with anti-tumor activity owing to their cytostatic and pro-apoptotic properties in multiple human cancer cell lines including meningioma cells [9], leukemia cells [10], hepatoma cells [11], melanoma cells, fibrosarcoma cells [12], colon cancer cells [13], and prostate cancer cells [14-16]. Some of the effects of frankincense essential oil were found to be related to the activities of sesquiterpenes and diterpenes [17].

Sandalwood (known as *Tan Xiang* in Chinese) belongs to the genus *Santalum*. The sandalwood essential oil has been used to treat skin diseases, acne, dysentery, and gonorrhea [18], and is considered an excellent sedating agent [19]. Sandalwood essential oil also exhibits anti-bactericidal activity [20] and chemoprevention in chemically induced skin papillomas and skin cancer in CD1 mice [21,22].

The genus *Boswellia* consists of four major species, while the genus *Santalum* consists of approximately 25 species. Each species of *Boswellia* and *Santalum* produces a slightly different type of aroma as a result of the soil and climate diversity. Frankincense and sandalwood essential oils are prepared from hydro- or steam-distillation of the plants [23]. Medical applications of essential oils ranging from treatments for skin conditions to remedies for cancer have been based on the historical uses of these plant products. However, the active chemical compositions and mechanisms of action remain largely unclear. We previously showed that frankincense essential oil prepared from *Boswellia* species with different temperatures and durations of hydrodistillation possessed different chemical constituents and biological activities [7,8].

This study aims to investigate the anti-proliferative and pro-apoptotic activities of frankincense and sandalwood essential oils on cultured human bladder cancer cells using microarrays and bioinformatics. We also intended to relate the cellular activity and gene expression profile to the chemical differences between frankincense and sandalwood essential oils.

## Methods

### Reagents and chemicals

Cell culture medium (MEM and DMEM/F-12 (1:1)), fetal bovine serum (FBS), MEM vitamin solution, non-essential amino acids, epidermal growth factor (EGF), insulin-transferrin-sodium selenite (ITS) media supplement, sodium pyruvate, and penicillin-streptomycin were purchased from Life Technologies (Grand Island, NY, USA). Frankincense (*B. carterii* from Somalia) and sandalwood (*S. album* from Sri Lanka) essential oils were obtained from Young Living Essential Oils (Lehi, UT, USA) and prepared based on previously reported procedures [7]. An XTT cell proliferation assay kit was obtained from Roche Applied Science (Indianapolis, IN, USA). An RNeasy® Mini Kit was obtained from Qiagen (Valencia, CA, USA).

### Chemical compositions of essential oils

The essential oil components were identified by gas chromatography–mass spectrometry (GC-MS), using an Agilent 7890A GC system (Agilent Technologies, Santa Clara, CA, USA) equipped with an Agilent 5975C mass selective detector (Agilent Technologies). We used either an HP-1 50 m × 0.32 mm ID × 0.5 μm film column (Agilent Technologies) or a DB-WAX 60 m × 0.32 mm ID × 0.5 μm film column (Agilent Technologies) for sandalwood essential oil, and an Rxi-5 ms 60 m × 0.25 mm ID × 0.25 μm column (Restek, Bellefonte, PA, USA) for frankincense essential oil. Retention indices were calculated by performing injections of C7-C30 normal alkanes (Supelco, Bellefonte, PA, USA) to confirm the MS identification. The detailed procedures for chemical analysis were reported previously [24].

As natural products, chemical constituents of frankincense and sandalwood essential oils varied from batch to batch and from season to season. A batch of distillation was used to establish a baseline relationship between chemical compositions and molecular pathways activation throughout the study.

### Human bladder cell lines

Human bladder cancer J82 cells, derived from a poorly differentiated, invasive stage 3 transitional cell carcinoma [25], were obtained from ATCC (HTB-1; Manassas, VA, USA). J82 cells were maintained in growth medium consisting of MEM supplemented with 10% FBS, 0.1 mM non-essential amino acids, 1 mM sodium pyruvate, 2% MEM vitamin solution, and 100 units/mL penicillin-100 μg/mL streptomycin. The UROtsa cell line was isolated from a primary culture of normal human urothelium and immortalized with the SV40 large T antigen [26]. UROtsa cells were provided by Dr. Ricardo Saban (Department of Physiology, University of Oklahoma Health Sciences Center) and cultured in DMEM/F12 supplemented with 10 ng/mL EGF, 1 × ITS media supplement, and penicillin-streptomycin. Both cell lines were maintained in a humidified cell incubator at 37°C and 5% CO_2_ and passaged every 3–4 days or when cells reached about 80% confluence.

### Cell viability assay

Bladder cancer J82 cells and immortalized normal UROtsa cells were seeded in 96-well tissue culture plates at a density of 5 × 10^3^ cells/well in 100 μL of their growth media to determine cell viability following treatment with frankincense and sandalwood essential oils. Following overnight incubation and adherence, an additional aliquot of growth media (100 μL) or varying dilutions of frankincense (1,400–600) (v/v) or sandalwood (16,000–7,000) (v/v) essential oil in the growth media was added to each well in triplicate. Measurement of cell viability following exposure to essential oils was performed using the XTT assay at time 0 and 24 h. During the assay, the growth medium (100 μL) was removed from each well and the XTT labeling mixture (50 μL) was added to each well. The reactions were carried out at 37°C for 4 h and the absorbance of the reaction product was recorded at 450 nm by a μQuant microplate reader (Bio-Tek; Winooski, VT, USA). Absorbance values obtained at 24 h following treatment were normalized to the values obtained at time 0 to calculate fold changes in cell viability [6].

### RNA extraction and quality evaluation

Total RNA was isolated from J82 cells for microarray analysis to determine frankincense and sandalwood essential oil-regulated molecular responses at the mRNA level. Briefly, 5 × 10^5^ J82 cells were seeded in 60-mm tissue culture plates, cultured overnight for adherence, and either left untreated or treated with a 1:1,100 dilution (v/v) of frankincense essential oil or a 1:11,000 (v/v) dilution of sandalwood essential oil in growth medium. Total RNA was isolated at 0 h (no treatment) and at 0.5, 1, 2, and 3 h after treatment using the RNeasy® Mini total RNA isolation kit. Total RNA concentration was determined using a nanodrop scanning spectrophotometer, and RNA was qualitatively assessed for degradation based on the ratio of 28S:18S rRNA using a capillary gel electrophoresis system (Agilent 2100 Bioanalyzer, Agilent Technologies).

### Probe synthesis and hybridization

Fluorescence-labeled probes were prepared from an aliquot (250 ng) of total RNA from each time point following essential oil treatments. First strand cDNA was reverse transcribed from total RNA in T7-oligo-dT; and cRNA was synthesized by *in vitro* transcription from the T7 promoter and labeled with biotinylated UTP by the Illumina Total Prep RNA Amplification Kit (Ambion; Austin. TX, USA). The biotinylatedlabeled cRNA probes were hybridized overnight to Illumina human Ref-8 version 3 BeadChips containing probes representating a total of 24,526 transcripts (Illumina, San Diego, CA, USA). Following stringent washes, microarray chips were incubated with streptavidin-Cy3 (Amersham Biosciences; Piscataway, NJ, USA) and scanned on an Illumina BeadArray Reader (Illumina).

### Microarray data normalization and transformation

Normalization of the microarray datasets was performed for each array by plotting a frequency histogram of the raw expression data for all genes [27,28]. The histograms showed a right-skewed unimodal distribution curve with the mode around zero. A normal distribution curve representing the genes with low levels of expression was then fitted around the mode, mirroring the Gaussian profile of the left part of the histogram. Two parameters mean and standard deviation (SD), were defined for this distribution. The expression values of the rest of genes were then normalized to the SD of the normal distribution curve after subtraction of the mean, and log10-transformed. The arrays were then adjusted to each other by robust linear regression [29,30]. Genes with expression value < 3.0 (or 0.477 on the log10 scale) were considered to be not expressed; this was equivalent to setting a threshold at three SD above the mean of low levels expression genes.

### Identification and clustering of hypervariable (HV) genes across the time course

We defined genes with statistically significant expression levels across the time points when comparing to a reference group [31] as HV genes. The reference group consisted of genes expressed above background levels but with low variability as determined by an F-test, and represented the technical variability of microarray data [27,32]. Genes whose expression levels varied significantly (*P* < 1/N, where N is the number of genes expressed above the background) were identified by comparing an individual gene’s variability to that of the reference group using an F-test [27]. The threshold level at *P* < 1/N is a modification of the Bonferroni correction for multiple hypothesis tests. These genes were further filtered to remove those with variability arising from experimental errors by comparing the variability of the residuals in the replicated group of samples with the same variability obtained after excluding the maximum and minimum, one at a time, using a modification of the ‘leave one out’ method [33]. A statistically significant decrease in variability after excluding one replicate provided evidence of possible error in that particular replicate. Once filtered, the remaining genes were denoted as HV genes [34], and were considered to be a snapshot of the dynamic biological responses following treatment with frankincense and sandalwood essential oils. The HV genes were mean-centered and clustered by hierarchical clustering using the ‘Correlation (uncentered)’ similarity metric. All statistical analyses and clustering were performed in Matlab (Natick, MA, USA).

### Ontology and canonical pathway analysis

Significantly over-represented gene ontologies for the identified HV genes were analyzed using the Database for Annotation, Visualization and Integrated Discovery (DAVID, http://david.abcc.ncifcrf.gov/) [35]. The genes of interest were uploaded as Illumina Probe IDs to DAVID; and the Illumina HUMANREF-8_V3_0_R1_11282963_A array was selected as a background. Functional annotation clustering was performed with default settings and medium stringency. Statistically significant over-representation of gene ontologies was reflected by the “enrichment score”; details of its calculation can be found on the DAVID website. Gene lists were analyzed for over-represented canonical pathways using Ingenuity Pathway Analysis (IPA; Ingenuity Systems, Redwood City, CA). Genes of interest were bound into a network using the “path explorer” tool, and the network was edited using the “path designer” tool in IPA.

### Semi-quantitative and quantitative reverse transcription (RT)-polymerase chain reaction (PCR) analysis

Total RNA samples were isolated from J82 cells treated with frankincense and sandalwood essential oils for RT-PCR. For semi-quantitative analysis, first-strand cDNA was synthesized from 2.5 μg of the total RNA with oligo (dT)_12–18_ primers (1 μg) (Life Technologies) and MMLV reverse transcriptase (200 U) (Life Technologies) in a total volume of 50 μL; the reaction was performed at 42°C for 2 h. Target mRNA species were amplified using a three-temperature cycle protocol: 94°C for 1 min, 55°C for 1 min, and 72°C for 1 min. PCR amplified products were separated on 1.5% agarose gels (Lonza, Walkersville, MD, USA) and stained with 0.5 μg/mL ethidium bromide (Life Technologies). Ethidium bromide-stained images were captured using a Gel Doc 1000 imaging system equipped with Quantity One® image analysis software (Bio-Rad Laboratories; Hercules, CA, USA).

SYBR Green-based quantitative PCR was used for quantifying the expression levels of target genes that are either commonly or differentially regulated by frankincense and sandalwood essential oils. Isolated total RNA was subjected to RNase-free DNase I (Qiagen, Valencia, CA, USA) (1 unit) digestion. PCR was performed using the above described three-temperature cycle with 30 s for each temperature reaction using a Bio-Rad iCycler real-time PCR detection system (Bio-Rad Laboratories) Melt-curve analysis was conducted after the final cycle to ensure the amplification of single species of PCR products in each sample; and agarose gel electrophoresis was used to confirm the presence of a single PCR band with the expected size in each reaction. We used β-actin as a reference gene to correct for sample-to-sample variations. Relative changes in JUN, DUSP1, PMAIP1, and ZNF311 expression after frankincense and sandalwood essential oils treatments were calculated based on Livak *et al.*[36].

### Statistical analysis

The results were expressed as means (SD) from at least 3 repeats. Comparisons of cell viability following frankincense oil treatment were made using one-way analysis of variance followed by a post hoc Dunnett's test. The Student’s *t*-test was used to compare levels of target gene expression between frankincense and sandalwood essential oil-treated J82 cells. *P* < 0.05 was considered statistically significant.

## Results

### Identification of frankincense and sandalwood essential oil chemical components

Frankincense and sandalwood essential oils were characterized by polarimetry and GC-MS. One of the best chemical signatures for *Boswellia carterii* is *alpha*-pinene ranging between 33.9% and 62.5% with a distinctive negative optical rotation (−13.3° ± 4.9°) as we previously reported [24].

Sandalwood essential oil (n = 23) exhibited unique chemical compounds *cis*-alpha-santalol and *cis*-beta-santalol at high concentrations, 41.1–47.9% and 15.9–21.3%, respectively. GC-MS with nonpolar HP-1 and polar DB-WAX qualified the purity of sandalwood essential oil as the method separated these two chemical components from similar compounds [37]. It is extremely difficult to establish standards for natural products. Even though standard operating procedures are established for harvest, storage, and processing, variable including weather, soil condition, and water quality all have significant impacts on the chemical compositions of all herbal products (*i.e.*, essential oils in this case). Therefore, this industry and scholarly publications present the chemical compositions of essential oils using a percentage range. However, with the introduction of analytical chemistry and molecular biology, we would like to relate and translate such descriptions of chemical compositions into descriptions of molecular responses, starting with the present article. Any deviation in molecular responses from the current observations will be related to changes in chemical compositions in the future. We hope that we will be able to create a “standard” for medical applications of essential oils or for all herbal products in the future.

### Tumor cell-selective versus non-selective cytotoxicity

**Figure 1:**
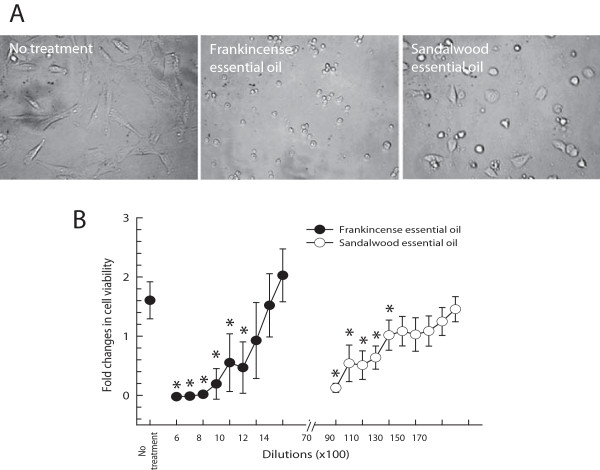
**Frankincense and sandalwood essential oils-suppressed viability in human bladder cancer J82 cells. (A)** Morphological changes of human bladder cancer J82 cells following frankincense (1:1,100 dilution) and sandalwood (1:11,000 dilution) essential oils treatment. Images were taken before and at 24 h following treatments by Olympus IX51 inverted microscope. **(B)** Cell viability determined by the XTT cell proliferation assay. Results were presented as mean (SD) from 4 independent experiments. *indicates statistical difference between untreated and essential oils-treated J82 cells (*P* < 0.05).

Both frankincense and sandalwood essential oils suppressed the viability of human bladder cancer J82 cells but with different morphologies (Figure 1A). At high concentrations of both essential oils (low dilution factors), no viable cells were detected, while frankincense and sandalwood essential oils significantly suppressed J82 cell viability at 1:1,100 (*P* = 0.008) and 1:11,000 (*P* = 0.012) dilutions (v/v), respectively (Figure 1B).

**Figure 2:**
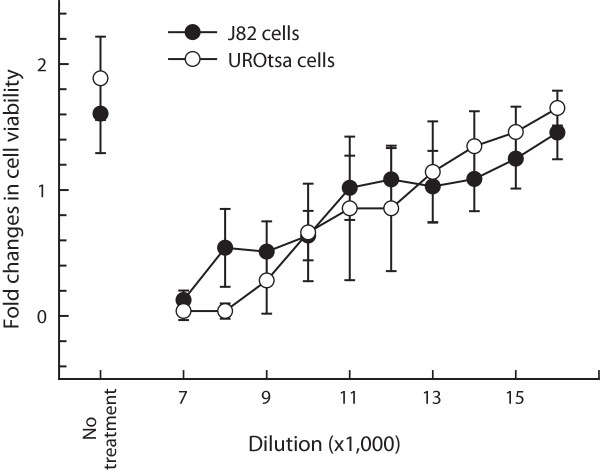
**Sandalwood essential oil-suppressed viability of J82 cells and immortalized normal bladder urothelial cells.** Data were presented as mean (SD) from 4 independent experiments.

Based on the results of cell viability assays, J82 cells were more sensitive than UROtsa cells to frankincense essential oil-suppressed cell viability, similar to the findings in our previous study [6]; the IC_50_ values were 1:1,250 and 1:600 dilutions (v/v) for J82 cells and UROtsa cells, respectively. In contrast, both J82 and UROtsa cells responded similarly to sandalwood essential oil treatment, with an IC_50_ value around 11,000 dilutions (v/v) for both cell lines (Figure 2).

### Identification of HV genes in frankincense and sandalwood essential oil-treated J82 cells

Temporal changes in gene expression patterns were analyzed in total RNA samples isolated from untreated J82 cells and cells that received either frankincense or sandalwood essential oil for between 0.5 and 3 h. Full microarray data have been deposited in Gene Expression Omnibus (GEO) with the accession number GSE53171, and are accessible on the GEO web-site (http://www.ncbi.nlm.nih.gov/geo/query/acc.cgi?acc=GSE53171).

**Figure 3:**
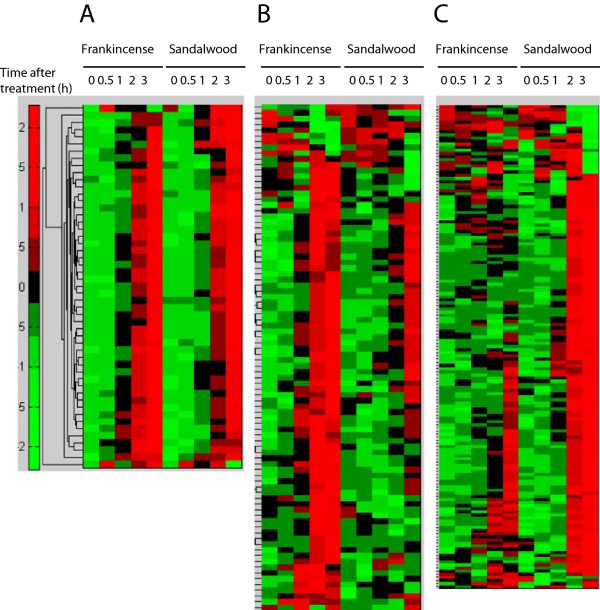
**Hierarchical clustering of HV genes-regulated by frankincense and sandalwood essential oils in bladder cancer J82 cells. (A)** Temporal expression profiles of genes that were commonly regulated by both frankincense and sandalwood essential oils. **(B)** Genes that were identified to be specifically regulated by frankincense essential oil. **(C)** Genes that were identified to be specifically regulated by sandalwood essential oil. Each column represents time intervals following essential oils exposure; and each row represents a gene probe. Expression levels for individual genes were scaled by green or red color indicating an elevated or suppressed level of expression, respectively.

A total of 139 genes were identified to be HV genes in frankincense essential oil-treated cells, whereas 206 HV genes were identified to be regulated by sandalwood essential oil (Additional file 1: Table S1). Among the HV genes, 51 genes were commonly regulated by both essential oils with a gradual increase in their expression levels over the experimental period (Figure 3A). Although the remaining HV genes also showed a gradual increase following stimulation with both essential oils, these genes responded differently between frankincense and sandalwood essential oils. A total of 88 HV genes were specifically regulated by frankincense essential oil, but their expression levels remained relatively constant following sandalwood essential oil treatment (Figure 3B). Another 155 HV genes were classified as sandalwood essential oil-responsive genes (Figure 3C).

### Ontological comparison of essential oil-regulated genes

The 139 and 206 HV genes regulated by frankincense and sandalwood essential oils, respectively, were subjected to DAVID analysis with Gene Ontologies (GO) of the over-represented HV genes. Four groups of genes were categorized into the GO terms of transcription factor activity, histone genes, negative regulation of biological process, and apoptosis (Table 1). Transcription factor activity was the most significantly over-represented class of genes regulated in frankincense and sandalwood essential oil-treated J82 cells. The expression of some transcription factors, such as ATF3, DDIT3, EGR1, FOSB, JUN, JUNB, and MYC, was modulated by both essential oils. Other transcription factors such as FOS (Finkel–Biskis–Jinkins murine osteogenic sarcoma virus) were specifically modulated by frankincense essential oil, whereas sandalwood essential oil modulated the expression of several inhibitors of DNA binding (ID1, ID2, and ID3), along with members of the zinc finger family. In addition, all identified histone core genes, except for HIST1H3D, were commonly modulated by both essential oils.

**Table 1:** Ontologies of frankincense and sandalwood essential oils-regulated genes in J82 cells

**Ontology**	**Genes specifically regulated by frankincense essential oil**	**Genes commonly regulated by frankincense and sandalwood essential oils**	**Genes specifically regulated by sandalwood essential oil**
Transcription factor activity	AHR, CEBPD, FOS, MXD1, NFAT5, RAXL1	ATF3, BHLHB2, DDIT3, EGR1, FOSB, JUN, JUNB, KLF10, KLF2, KLF4, MYC, TSC22D1	CEBPB, CITED4, DSCR1, ENO3, HES1, HLX1, ID1, ID2, ID3, IRF1, KLF5, KLF6, NFIL3, SIX4, SMAD7, TTRAP, ZNF131, ZNF165, ZNF175, ZNF18, ZNF256, ZNF483
(1.90E-05 - 5.70E-04)			
Histone core	HIST1H3D	HIST1H2AC, HIST1H2AM, HIST1H2BF, HIST1H4E, HIST2H2AA3, HIST2H2AC	
(3.30E-07 - 4.50E-05)			
Negative regulation of biological process	BIRC3, HSPA1B, NFKBIL2, NUAK2, SPTBN1, UHMK1	BHLHB2, CDKN1A, DDIT3, DDIT4, EGR1, FOSB, HES1, ID1, IL1A, IL6, IL8, JUN, KLF10, KLF4, MYC, PIM1, RGS2, TNFAIP3, ZFP36	ANGPTL4, CEBPB, DLC1, ENO3, GADD45A, HMOX1, ID2, ID3, IER3, ING5, IRF1, LEP, MCL1, PMP22, PPP1R15A, PTHLH, TRIM13
(8.00E-06 - 4.50E-07)			
Apoptosis	AHR, BIRC3, HSPA1B, NGFRAP1, NUAK2, NUDT2, PRKDC	AXUD1, CDKN1A, DDIT3, DDIT4, IL1A, IL6, PHLDA1, PIM1, SGK, TNFAIP3	ANGPTL4, C8orf4, CASP6, CEBPB, GADD45A, GADD45B, HMOX1, ID3, IER3, MCL1, PMAIP1, PPP1R15A, ZMAT3
(4.60E-04 - 1.70E-04)			

Negative regulation of biological processes categorized by IPA was more pronounced in sandalwood essential oil-treated J82 cells. Among the most notable HV genes, growth arrest genes (GADD45A, GADD45B, and PPP1R15A) and pro-apoptotic genes (CASP9 and ING5) were specifically up-regulated by sandalwood essential oil. As part of the negative regulation of biological processes, several pro-inflammatory interleukins (IL1A, IL6, and IL8) were up-regulated by both essential oils.

### Specific genes modulated by frankincense or sandalwood essential oil

The 51 genes commonly regulated by both essential oils were excluded to identify over-represented genes specifically modulated by either frankincense or sandalwood essential oil in J82 cells. The remaining 88 and 155 HV genes specifically modulated by frankincense and sandalwood essential oils, respectively (Figure 3B and C), were analyzed by DAVID. Heat shock proteins (HSP), including three members of DNAJ family (HSP40 homolog), were the main over-represented call of HV genes following frankincense essential oil treatment (Table 2). In addition to the histone core family genes (Table 1), three other members of this family were significantly over-represented among the HV genes identified in frankincense essential oil-treated J82 cells.

**Table 2:** Ontologies unique to frankincense or sandalwood essential oil-regulated genes

**Ontologies**	**Genes**	**Enrichment score**
**Frankincense essential oil**		
Heat shock protein DNAJ	DNAJA4, DNAJB1, DNAJB4	1.59
Histone core	HIST1H2BD, HIST1H3D, HIST2H4A	1.23
DNA-binding	AHR, CCNL1, CEBPD, FLJ23436, FOS, MXD1, NFAT5, PHC3, POLE, RAXL1, ZFAND2A, ZFP91, ZNF654	0.68
Protein kinase activity	ABL2, EPHB4, NUAK2, PDK4	0.53
**Sandalwood essential oil**		
Transcription regulation	48 genes, predominantly zinc finger proteins	12.25
Negative regulation of protein kinase activity	DUSP6, GADD45B, SPRY4	0.78
Cell death	EMP1, IER3, MYADM	0.28
Protein kinase activity	MAP3K8, RAGE, SCYL3	0.18
G-protein coupled receptor	FCAR, GPR1, PTGER4, CLDN1, GPR154	0.01

Transcriptional regulation was identified as the most over-represented class of genes that were specifically modulated by sandalwood essential oil in J82 cells. Among these transcription factors, members of the zinc finger family of proteins were predominantly identified (Table 2). In addition, cell death-related genes were over-represented in sandalwood essential oil-treated cells, specifically epithelial membrane protein 1, immediate early response 3, and myeloid-associated differentiation marker.

### Canonical pathway comparison of genes modulated by frankincense and sandalwood essential oils

**Figure 4:**
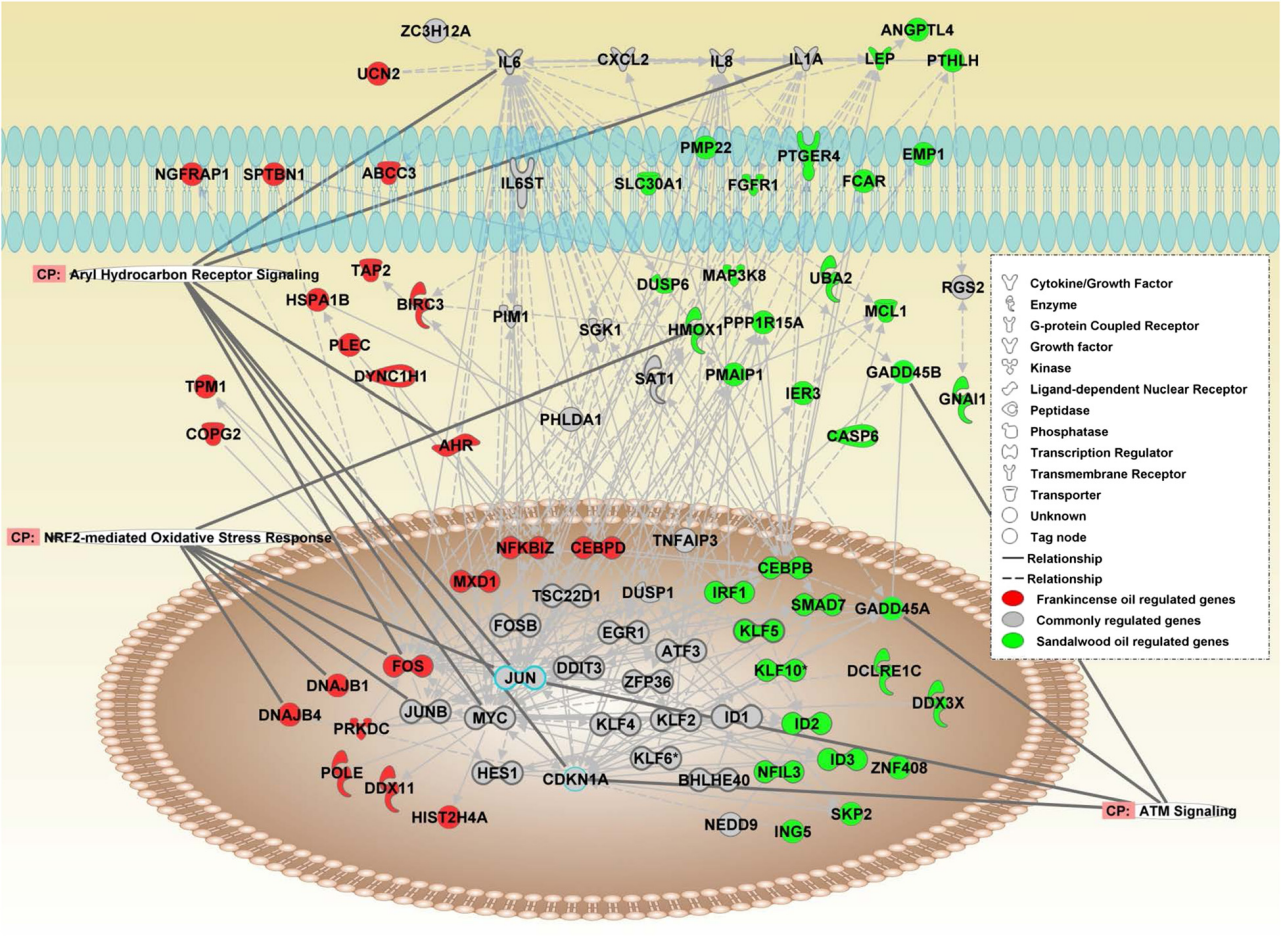
**Frankincense and sandalwood essential oils-activated gene networks in bladder cancer J82 cells.** Gene networks were composited by HV genes that were regulated **(A)** specifically by frankincense essential oil (red), **(B)** specifically by sandalwood essential oil (green), or **(C)** commonly by both frankincense and sandalwood essential oils (gray). Identified genes that belong to definite biological processes were highlighted.

The HV genes modulated by frankincense and sandalwood essential oil treatments in J82 cells were analyzed by IPA to identify canonical pathways through connecting both essential oil-modulated genes in a single network (Figure 4). Consistently, cell death genes (67/84 genes, *P* = 8.27E-33) and apoptotic genes (62/84 genes, *P* =1.69E32) represented significantly enriched ontological categories.

Pathways including the p38 MAPK, p53, IL6, and HMGB1 signaling pathways were commonly represented by HV genes modulated by both essential oils (Table 3). IPA also identified canonical pathways that were specific for either frankincense or sandalwood essential oil treatment. For example, aryl hydrocarbon receptor (AhR) signaling and NRF2-mediated oxidative stress response genes were the two main canonical pathways over-represented by the HV genes regulated by frankincense essential oil. In contrast, ATM signaling was uniquely over-represented by the HV genes identified in J82 cells treated with sandalwood essential oil.

**Table 3:** Canonical pathways induced by frankincense and sandalwood essential oils treatment

**Canonical pathways**	**Unique to frankincense oil**	**Common genes**	**Unique to sandalwood oil**
Aryl hydrocarbon receptor signaling (6.61E-05)	AHR, CDKN1A, FOS, IL1A, IL6, JUN, MYC	---	---
NRF2-mediated oxidative stress response (1.58E-03)	DNAJA4, DNAJB1, DNAJB4, FOS, JUN, JUNB	---	---
ATM signaling (1.74E-05)	---	---	CDKN1A, GADD45A, GADD45B, JUN, RAD50, TLK1
p38 MAPK signaling (6.92E-06 - 7.94E-05)	JMJD7-PLA2G4B	DDIT3, DUSP1, DUSP10, IL1A, IRAK2, MYC	HIST2H3D
p53 signaling (2.75E-02 - 3.98E-04)	PRKDC	CDKN1A, JUN	CASP6, GADD45A, GADD45B, PMAIP1
IL-6 signaling (5.25E-05 - 4.17E-04)	FOS	IL1A, IL6, IL6ST, IL8, JUN	CEBPB
HMGB1 signaling (5.89E-04 - 3.16E-03)	FOS	IL1A, IL8, JUN, RND3	RAGE

### Analysis of transcript expression by RT-PCR

**Figure 5:**
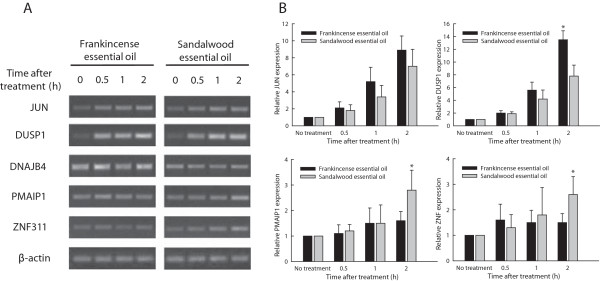
**Confirmation of genes expression identified in microarray by RT-PCR.** Limited number of genes identified to be regulated by frankincense, sandalwood essential oils or both from microarray results were confirmed by RT-PCR. **(A)** Representative images of RT-PCR amplified JUN, DUSP1, DNAJB4, PMAIP1, ZNF311, and β-actin from frankincense and sandalwood essential oils-treated J82 cells were presented. **(B)** Relative changes of JUN, DUSP1, PMAIP1, and ZNF311 transcripts expression were determined by quantitative RT-PCR analysis. Data were presented as mean (SD) from at least 3 independent experiments.

Microarray and RT-PCR analysis provided consistent results for these genes. Although both JUN and DUSP1 were up-regulated by both essential oils in J82 cells (Figure 5A), the levels of DUSP1 were significantly elevated in frankincense essential oil-treated cells (*P* = 0.011), whereas there was no statistical significance for JUN (*P* = 0.282) at 2 h following treatment (Figure 5B). DNAJB4 was up-regulated by frankincense essential oil (*P* = 0.043), whereas PMAIP1 (*P* = 0.032) and ZNF311 (*P* = 0.035) transcripts were specifically up-regulated in sandalwood essential oil-treated J82 cells at 2 h after stimulation.

## Discussion

In this study, we generated GC-MS profiles for hydrodistilled frankincense (*B. carterii* from Somalia) and steam-distilled sandalwood (*S. album* from Sri Lanka) essential oils and compared their anti-cancer activities in human bladder cancer J82 and immortalized normal human urothelial UROtsa cell lines.

A genome-wide gene expression analysis differentiated the genes and pathways that were modulated by frankincense and sandalwood essential oils in J82 cells. We compared comprehensive transcriptome expression patterns between frankincense and sandalwood essential oils based on their distinctive chemical compositions. Sandalwood essential oil contains the highest percentages of hydroxylated sesquiterpenes, such as santalols (47–56%) [38]; frankincense essential oil consists of high percentages of monoterpenes, such as *alpha*-pinene (60–80%) [7,24].

These two essential oils had different chemical profiles [24,39], but elicited similar cellular responses leading to activation of activator protein 1 (AP-1). AP-1 activation can be induced by environmental stresses, growth factors, and cytokines [40-42], and is implicated in cell proliferation, differentiation, apoptosis, and transformation [43,44]. Several pro-inflammatory cytokines, including IL1A, IL6, and IL8, commonly regulated by both essential oils, have been implicated in the p38 MAPK pathway and might be responsible for subsequent AP-1 activation [45]. However, differential expression of AP-1 components following essential oil exposure was observed. Several AP-1 subunits, FOSB, JUN, and JUNB, were regulated by both frankincense and sandalwood essential oils, while FOS expression was specifically affected by frankincense essential oil. The composition of the AP-1 heterodimer might determine the AP-1-regulated proliferative or apoptotic activities [44]. Unique FOS regulation by frankincense essential oil might cause differential effects of AP-1 activation. FOS forms stable heterodimers with JUN and enhances AP-1 transcriptional capacity [46], but is not required for apoptosis [47]. The AP-1 stability owing to FOS expression following frankincense essential oil treatment might protect normal cells against the essential oil’s cytotoxicity.

In addition to AP-1 transcription factor, EGR1 is another transcription factor that is involved in the negative regulation of biological processes [48]. Other transcription factors, such as CEBPB, ENO3, ID2, ID3, and IRF1, specifically regulated by sandalwood essential oil, are negative regulators of biological processes, suggesting that sandalwood essential oil suppresses cancer cell viability *via* an array of multiple transcription factors activities. A large number of histone core genes were regulated by both frankincense and sandalwood essential oils, suggesting that both essential oils alter chromosome structure and the accessibility of DNA to transcription factors.

MYC expression was up-regulated by both frankincense and sandalwood essential oils in J82 cells. Although MYC sensitized cells to undergo apoptosis [49], frankincense essential oil up-regulated a MYC partner MXD1 (MAX dimerization protein 1, or MAD). MXD1 might antagonize MYC transcriptional activity by forming a DNA-binding complex with MAX in the core sequence 5'-CAC[GA]TG-3’ [49].

Aryl hydrocarbon signaling was specifically overrepresented in J82 cells treated with frankincense essential oil, as evidenced by the up-regulation of AhR mRNA. Activated AhR regulates downstream gene expression, including xenobiotic metabolizing enzymes and phase II metabolizing enzymes, as well as growth factors and p53 target genes such as p21CIP1 (CDKN1A). Activation of AhR signaling may be a consequence of an imbalance between the production of reactive oxygen and the detoxification of reactive intermediates in frankincense essential oil-treated J82 cells, as reflected by possible activation of the NRF2-mediated oxidative stress response pathway. Two regulators of NRF2 transcription factor, FOS and JUN, as well as NRF2-regulated stress response genes (DNAJ heat shock proteins), were specifically up-regulated by frankincense essential oil. The selective cancer cell death induced by frankincense essential oil could be a result of oxidative stress.

The effect on ataxia telangiectasia mutated (ATM) signaling was unique to sandalwood essential oil-treated J82 cells. ATM protein is a key regulator of multiple signaling cascades following damage and subsequent DNA repair. The expression of RAD50 was up-regulated in this study, and RAD50 might activate ATM. RAD50 protein is involved in DNA double-strand break repair; and its expression might be a result of DNA damage induced by sandalwood essential oil. The ATM activation was evidenced by up-regulations of multiple p53 substrates, including the GADD45 (growth arrest and DNA damage) complex and CDKN1A. Up-regulation of GADD45 and CDKN1A, which can lead to cell cycle arrest at the G2/M checkpoint with subsequent apoptosis [50,51], was observed in sandalwood essential oil-treated J82 cells. Additionally, the involvement of p53 activation in sandalwood essential oil-treated J82 cells was evidenced by CASP6 and PMAIP1 (NOXA) expression in the apoptosis branch of the p53 pathway. The sandalwood essential oil-induced cytotoxicity might be a result of DNA instability, chromatin remodeling, and DNA double stranded breaks.

High mobility group box-1 (HMGB1) signaling, which is an integral component of oxidative stress and downstream cell survival or death, including apoptosis and autophagy [52,53], was identified as a common canonical pathway activated by both frankincense and sandalwood essential oils. HMGB1 is involved in the assembly of nucleoprotein complexes to maintain nucleosome structure and regulate gene transcription, and is secreted by cells after stimulation with endotoxins and cytokines, including IL1, IL6, or IL8 [54]. HMGB1 might act synergistically with oxidative stress induced by frankincense essential oil. The expression of the receptor for advanced glycation end products (RAGE) that binds to HMGB1 [55] was specifically regulated in sandalwood essential oil-treated cells. HMGB1 has been demonstrated to induce apoptosis *via* the RAGE-p38 MAPK/ERK signaling pathway [56]. Thus, sandalwood essential oil-induced HMGB1 signaling might occur synergistically with p38 MAPK signaling through the RAGE receptor and result in non-selective cell death.

Frankincense essential oil may represent a candidate on a growing list of natural compounds selectively eradicating cancer cells *via* oxidative stress [57-59]. To our knowledge, this is the first report suggesting that the NRF2-mediated oxidative stress response pathway might be involved in the tumor cell-specific anti-proliferative and pro-apoptotic activities of frankincense essential oil. In contrast, sandalwood essential oil appears to be a non-tumor cell-specific agent inducing DNA damage and cell cycle arrest. Further studies are required to confirm the involvement of these pathways in relevant biological systems, *e.g.*, whether the redox status of HMGB1 induced by frankincense essential oil is involved in cancer cell-specific activity.

## Conclusion

The effects of frankincense and sandalwood essential oils on J82 cells and UROtsa cells involved different mechanisms leading to cancer cell death. While frankincense essential oil elicited selective cancer cell death *via* NRF-2-mediated oxidative stress, sandalwood essential oil induced non-selective cell death *via* DNA damage and cell cycle arrest.

## Abbreviations

AhR: Aryl hydrocarbon receptor; AP-1: Activation protein 1; ATM: Ataxia telangiectasia mutated; DAVID: Database for annotation, Visualization and Integrated discovery; EGF: Epidermal growth factor; GADD45: Growth arrest and DNA damage; GO: Gene ontology; HMGB1: High mobility group box-1; HSP: Heat shock protein; HV: Hypervariable; IER: Immediate early response; IPA: Ingenuity pathway analysis; RT: Reverse transcription; SD: Standard deviation; TCC: Transitional cell carcinoma; FOS: Finkel–Biskis–Jinkins murine osteogenic sarcoma virus.

## Competing interests

CW and GY are employed by the company Young Living Essential Oils. Other authors declare that they have no competing interests.

## Authors’ contributions

MS, CW, GY, KMF, and HKL conceived the study and designed the experiments. QY, WW, HYL, MD and JW performed the experiments. MS, CW, GY, KMF, HKL, MD and JW interpreted the experimental results. All authors prepared the manuscript preparations and approved the final manuscript.

## Supplementary Material

Additional file 1: Table S1 Genes that are regulated by frankincense and sandalwood essential oils in J82 cells. Click here for file
